# SPEEK and SPPO Blended Membranes for Proton Exchange Membrane Fuel Cells

**DOI:** 10.3390/membranes12030263

**Published:** 2022-02-25

**Authors:** Muhammad Imran Khan, Abdallah Shanableh, Shabnam Shahida, Mushtaq Hussain Lashari, Suryyia Manzoor, Javier Fernandez

**Affiliations:** 1 Research Institute of Sciences and Engineering (RISE), University of Sharjah, Sharjah 27272, United Arab Emirates; mimran@sharjah.ac.ae (M.I.K.); shanableh@sharjah.ac.ae (A.S.); 2 Department of Chemistry, University of Poonch, Rawalakot 12350, Pakistan; shabnamshahida01@gmail.com; 3 Department of Zoology, The Islamia University of Bahawalpur, Bahawalpur 63100, Pakistan; mushtaqlashary@gmail.com; 4 Institute of Chemical Sciences, Bahauddin Zakariya University, Multan 60800, Pakistan; suryyia.manzoor@bzu.edu.pk; 5 Department of Chemical Engineering, University College London, Torrington Place, London WC1E 7JE, UK; 6 IQS School of Engineering, Universitat Ramon Llull, Via Augusta, 390, 08017 Barcelona, Spain

**Keywords:** SPEEK, ion exchange membrane, redox flow battery, stability, proton conductivity, blended membranes, SPPO, fuel cell

## Abstract

In fuel cell applications, the proton exchange membrane (PEM) is the major component where the balance among dimensional stability, proton conductivity, and durability is a long-term trail. In this research, a series of blended SPEEK/SPPO membranes were designed by varying the amounts of sulfonated poly(ether ether ketone) (SPEEK) into sulfonated poly(phenylene) oxide (SPPO) for fuel cell application. Fourier transform infrared spectroscopy (FTIR) was used to confirm the successful synthesis of the blended membranes. Morphological features of the fabricated membranes were characterized by using scanning electron microscopy (SEM). Results showed that these membranes exhibited homogeneous structures. The fabricated blended membranes SPEEK/SPPO showed ion exchange capacity (IEC) of 1.23 to 2.0 mmol/g, water uptake (W_R_) of 22.92 to 64.57% and membrane swelling (MS) of 7.53 to 25.49%. The proton conductivity of these blended membranes was measured at different temperature. The proton conductivity and chemical stability of the prepared membranes were compared with commercial membrane Nafion 117 (Sigma-Aldrich, St. Louis, Missouri, United States) under same experimental conditions. The proton conductivity of the fabricated membranes increased by enhancing the amount of SPPO into the membrane matrix. Moreover, the proton conductivity of the fabricated membranes was investigated as a function of temperature. Results demonstrated that these membranes are good for applications in proton exchange membrane fuel cell (PEMFC).

## 1. Introduction

Fuel cells (FCs) serve to convert fuels into electric power in an efficient and environmentally friendly manner. Proton exchange membrane fuel cells (PEMFCs) are compact, lightweight with high current densities and hence are advantageous over other fuel cells [1,2,3]. Compared to other sources of energy, PEM fuel cells have many advantages. They act as eco-friendly renewable energy systems and can be stored for application as a single cell or many cells combined together, forming a cell stack to obtain higher voltage and electricity [4,5,6]. PEM fuel cells have advantage of burning in reduced temperature compared to other systems. Compared to other energy generating mediums, fuel produced from PEM fuel cells is considered clean and stable. They are reliable for a wide range of portable and stationary power uses. In addition to using PEM fuel cell as a standalone power generator, the PEM fuel cell can also be implemented with a renewable energy system for energy storage application [4,7,8,9].

PEMs serve as the prime components in polymer electrolyte membrane fuel cells (PEMFCs) and they have defined criteria for their applications in water uptake, proton conductivity, thermal, mechanical, and chemical stability [10]. Polyfluorosulfonate ionomer membranes such as Nafion membranes are the common commercial membranes employed in the PEMFC due to enhanced proton conductivity [11]. Nevertheless, application of these membranes is limited by high cost and narrow operational temperature range [12]. Moreover, a major barrier lies in the fabrication of PEMs that showed high chemical, electrochemical and thermal stability, low reactant permeability, improved proton conductivity and are cost effective [13,14,15,16].

A wide range of materials as a substitute for Nafion have been described. A major development in this regard is seen in the use of sulfonated aromatic polymer (sulfonated polyimide) [17], sulfonated poly ether sulfone (SPES) [18], sulfonated poly (phenylene oxide) (SPPO) [19] and sulfonated poly ether ether ketone (SPEEK) [6,20]. Recently, SPES and SPEEK have shown potential applications for direct methanol fuel cell due to their cost effectiveness and improved chemical stability [21,22,23,24]. However, SPEEK membranes suffered a substantial swelling ratio due to high degree of sulfonation which ultimately affects their mechanical strength [25]. Xi et al. reported the fabrication of SPEEK membranes by using SPEEK with different degree of sulfonation (DS) for redox flow batter applications [26]. Sun et al. reported the comparison of SPEEK membrane with Nafion membrane in redox flow battery [27]. Polymer blending is considered an effective procedure to alter and improve the membrane properties and performance [28,29]. However, it is also reported that the membranes fabricated by blending two kinds of sulfonated polymers resulted in high proton conductivity, good performance and miscible structure [30,31].

In order to improve the performance of PEMs, the blending of SPEEK and SPPO was proposed. Among the non-fluorinated membranes materials, sulfonated poly (ether ether ketone) is another ion polymer that has high mechanical and thermal stability along with good proton conductivity [32]. Some of the properties of individual polymer can be used as a basis to device the novel membranes with desirable performance by blending [33,34].

This work reports the fabrication of a series of SPEEK, SPPO and blended SPEEK/SPPO proton exchange membranes (PEMs) by varying the amount of SPPO into the polymer matrix. The effect of the amount of SPPO onto ion-exchange capacity (IEC), water uptake (W_R_), membrane swelling (MS), thermal, chemical and mechanical stability, and morphology were investigated in detail. The synthesis of these blended membranes was confirmed by FTIR spectroscopy. The proton conducting performance of these prepared blended membranes SPEEK/SPPO was investigated at different temperature and compared commercial membrane Nafion 117.

## 2. Experimental

### 2.1. Materials

Na^+^ form of SPPO was obtained from Tianwei Membrane Co., Ltd. (Shandong, China (ion exchange capacity: 2.0 mmol/g dry). N-methyl-2-pyrrolidolone (NMP, AR grade), sulfuric acid (98%), sodium chloride (AR grade) and sodium hydroxide (AR grade) were bought from Shanghai-Sinopharm Chemical Reagent Co. Ltd., Shanghai, China. Deionized (DI) water was used throughout this work.

### 2.2. Sulfonation of PEEK

**Figure 1:**
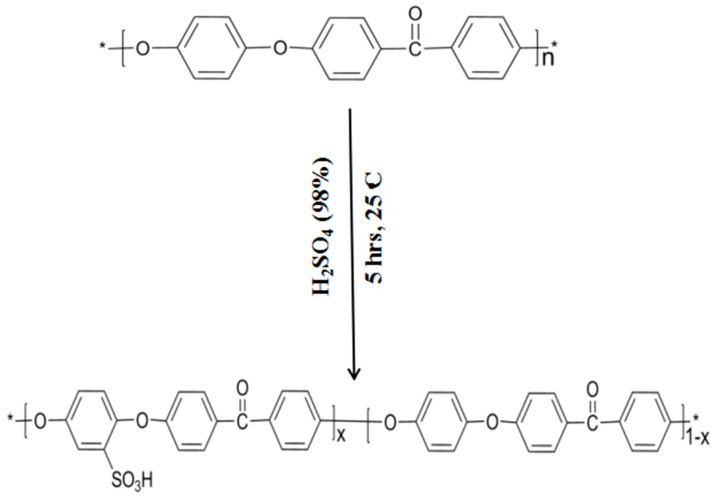
Sulfonation of PEEK.

Sulfonation of PEEK polymer was carried out following the method described in literature (Figure 1) [35]. Firstly, 5 g of PEEK was dissolved into concentrated H_2_SO_4_ (90 mL) and stirred for 5 h at room temperature. Next, the reaction mixture was added to a container with cold water. Sulfonated PEEK (SPEEK) was obtained in the form of white precipitate. The product obtained was neutralized by excessive washing with deionized water and dried in vacuum oven at 80 °C for 8 h. The final product was the sulfonated form of polyether ether ketone with a sulfonation degree of 16.0%.

### 2.3. Designing of the Blended Membranes

Solution casting method was used to fabricate the SPEEK/SPPO blended membranes as reported previously [36,37,38,39,40,41,42]. In a typical procedure, we got homogeneous solution of SPEEK by dissolving its calculated amount into 20 mL of N-Methyl-2-pyrrolidone (NMP). Then, the homogeneous solution of sulfonated poly (2,6-dimethyl-1,4-phenylene oxide) (SPPO) was obtained by dissolving the varying amounts of it into N-Methyl-2-pyrrolidone (NMP) solvent. To acquire five blended membranes with different physico-chemically, the concentration of SPPO was varied from 0 to 100% in order. This solution was added drop wise into the above prepared SPEEK solution. We named the obtained membranes as SPEEK/SPPO-0%, SPEEK/SPPO-25%, SPEEK/SPPO-50%, SPEEK/SPPO-75% and SPEEK/SPPO-100%, respectively, according to the concentration (%) of SPPO. The obtained solution was dispersed evenly by mean of mechanical stirring and then casted on the glass plate and placed in the oven at 70 °C to evaporate solvent.

### 2.4. Characterizations

#### 2.4.1. Instrumentations

Herein, we employed attenuated total reflectance (ATR) with FTIR spectrometer (Vector 22, Bruker, Massachusetts, MA, USA) in the range of 4000–400 cm^−1^ to confirm the fabrication of SPEEK/SPPO blended membranes. Tensile strength of the prepared blended membranes was investigated by using Q800 dynamic mechanical analyzer (DMA, TA Instruments, Kyoto Japan) at a stretch rate of 0.5 N/min. Morphology of these blended membranes was studied by employing field emission scanning electron microscope (FE-SEM, Sirion200, FEI Company, Hillsboro, OR, USA). Thermal stability was evaluated by using the Shimadzu TGA-50H analyzer (Kyoto, Japan) under nitrogen flow with a heating rate of 10 °C/min within the temperature range of 25 to 800 °C.

#### 2.4.2. Ion Exchange Capacity, Water Uptake and Membrane Swelling

Ion exchange capacity (IEC) describes the extent of interchangeable ionic groups (equivalents) present per dry membrane mass [43,44]. IEC was determined through classical Mohr’s method. Firstly, the prepared blended membranes SPEEK/SPPO were immersed into 1.0 (M) HCl solution for one day such that all charge sites were changed into the H^+^ form. The washing of blended membrane samples was then performed using deionized water to remove any excess of HCl. The washed membranes were then dipped for 2 days into 50 mL of 2 M NaCl solution. The amount of H^+^ ions liberated was measured through acid-base titration using standardized 0.01 M NaOH solution. Ion exchange capacity was calculated by using the following equation:

(1) $$IEC=\frac{ab}{w}$$

Here the dry weight of the membrane, titre volume during titration and the concentration of NaOH solution are denoted by *w* (g), *a* (mL) and *b* (mg/L), respectively.

Water uptake of ion exchange membrane (IEM) represents its hydrophilicity [45]. The fabricated membranes were firstly oven dried and accurately weighed. Then they were soaked in water for 2 days at room temperature. Wet weight of these membranes was then measured. Water uptake ability was determined by employing the following relationship [38,46,47]:

(2) $$W_{R}=\frac{W_{\mathrm{WET}}-W_{DRY}}{W_{DRY}}\times 100\%$$

where *W*_WET_ (g) and *W_DRY_* (g) are the weights of wet and dry membranes, respectively.

The membrane swelling (*MS*) was recorded by utilizing the dry and wet lengths of the fabricated blended membranes. *MS* of the fabricated blended membranes was determined by utilizing the following relationship [36,37]:

(3) $$MS(\%)=\frac{L_{w}{}_{}-L_{d}}{L_{d}}\times 100$$

where *L_d_* (cm) and *L_w_* (cm) are the lengths of dry and wet membranes, respectively.

#### 2.4.3. Chemical Stability of Membranes

The chemical stability test was investigated by soaking the dried blended membranes into the Fenton’s reagent (3 w% H_2_O_2_; 3 mg/L Fe^2+^) at 60 °C for different time intervals. Then, they were washed, dried and change in weight ratio was determined [48,49].

#### 2.4.4. Proton Conductivity Measurement

The frequency range of 10 Hz to 10 kHz on an electrochemical workstation (PARSTAT 2273, Princeton) was selected to determine the proton impedance at 80 °C and 100% relative humidity (RH). The membrane was immersed in 0.2 M H_2_SO_4_ solution for 24 h and then neutralized with distilled water. The proton conductivity (*σ*, mS/cm) was determined by using Equation (4):

(4) $$\sigma =\frac{l_{o}}{AR}$$

where *l_o_* (cm) represents the distance between the electrodes used to determine the potential, *A* (cm^2^) is the effective surface area and *R* (Ω) is the membrane impedance.

## 3. Results and Discussion

### 3.1. FTIR

**Figure 2:**
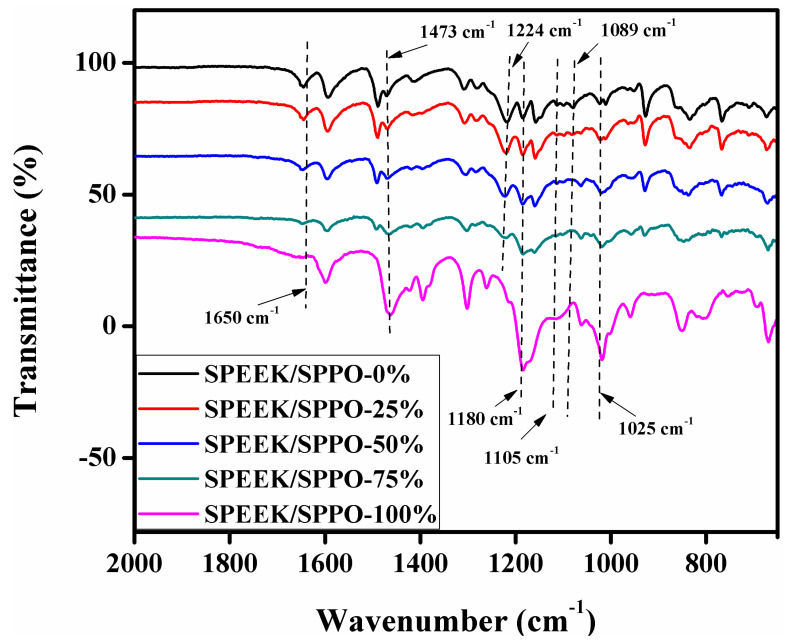
IR spectrums of SPEEK, SPPO and SPEEK/SPPO blended membranes (where 0, 25, 50, 75 and 100% represent the amount of SPPO into the SPEEK/SPPO blended membranes).

Figure 2 shows FTIR spectrums of SPEEK, SPPO and SPEEK/SPPO blended membranes. The three spectrums were similar apart from some specific peaks. The symmetric and asymmetric stretching vibrations of the sulfonic acid group for three membranes appeared at 1025 cm^−1^ and 1089 cm^−1^, respectively [50,51]. The intensities of these peaks are different for all the prepared blended membranes which represented the degree of sulfonation (DS) difference among these membranes. The peaks at 1105 cm^−1^ were due to aromatic ring. The peaks at 1473 cm^−1^ (C-C aromatic ring) and 1650 cm^−1^ (C=C aromatic ring) depicted specific bonds of SPEEK for SPPO and SPEEK/SPPO blended membranes. The –S=O stretching in the SPPO and SPEEK/SPPO blended membranes appeared at 1180 cm^−1^ [52]. Moreover, the peaks observed at 1224 cm^−1^ in the SPEEK and SPEEK/SPPO blended membranes corresponds to the aromatic structure (C-O-C) which was absent in SPPO membrane. It proved that SPEEK and SPPO were well blended into the SPEEK/SPPO membranes.

### 3.2. Morphology of Membranes

**Figure 3:**
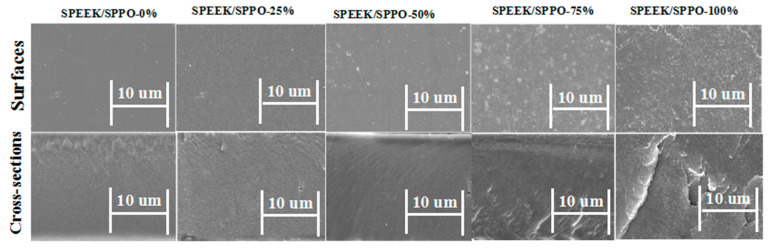
SEM images of surfaces and cross-sections of the membranes SPEEK/SPPO-0 to SPEEK/SPPO-100 (where 0, 25, 50, 75 and 100% represent the amount of SPPO into the SPEEK/SPPO blended membranes).

Herein, scanning electron microscopy (SEM) was utilized to investigate morphological features of surfaces and cross-sections of the fabricated SPEEK, SPPO and blended SPEEK/SPPO. Figure 3 is about SEM micrographs of the prepared SPEEK, SPPO and blended SPEEK/SPPO membranes. Results showed that all the membranes exhibited homogeneous morphologies. The surfaces and cross-sections of pristine SPEEK membrane were highly transparent. There was no hole in the prepared blended membranes except SPEEK/SPPO-25% and SPEEK/SPPO-100% membranes. The morphology of the blended membranes becomes rougher with the incorporation of SPPO into the polymer matrix as shown in Figure 3. Both the polymers contain hydrophilic group (-SO_3_H), indicating the good compatibility with each other [53]. Therefore, the blended membranes were prepared without any notable defects. Overall, all the prepared blended membranes represented homogeneous structure which is useful for proton conductivity of PEMs.

### 3.3. Ion Exchange Capacity (IEC)

*IEC* of an ion exchange membrane (IEM) suggests the density of ion exchangeable groups which serves as an indicator of proton transfer capacity. It depends on the density of sulfonic acid groups into the polymer matrix [54]. Table 1 denotes the measured *IEC* of the prepared blended membranes. *IEC* of the pristine SPPO membrane and blended SPEEK/SPPO membranes were greater than that of pristine SPEEK. *IEC* of the blended membranes also increased with increasing the quantity of SPPO due to improved proton conductivity.

### 3.4. Water Uptake and Membrane Swelling

Water uptake plays an important role in the proton transport from anode to cathode. Water molecules can support vehicle and Grotthus mechanism though providing proton carriers and forming hydrogen bond networks, respectively [55]. Water uptake of the prepared blended membranes was measured at room temperature and results are shown in Table 1. Water uptake of SPPO membrane and blended SPEEK/SPPO membranes were higher than that of pristine SPEEK membrane. The water uptake value for the fabricated blended membranes was increased with increasing the quantity of SPPO into the polymer matrix.

The membrane swelling (*MS*) is the commonly employed parameter to evaluate the dimensional stability of PEMs. Generally, a poor mechanical stability and low durability is observed in case of high membrane swelling. Table 1 shows *MS* properties. The *MS* of the fabricated blended membranes was increased from 7.53 to 25.49% with increasing *IEC* values when the amount of SPPO was increased into the polymer matrix.

### 3.5. Mechanical Stability

Table 2 represents tensile strength (TS) and elongation at break (*E_b_*) values of the fabricated SPEEK, SPPO, and blended SPEEK/SPPO. The value of TS was found to be 7.58 to 20.42 MPa while *E_b_* 3.89 to 41.91% for the fabricated membranes. From the fabricated membranes, the pristine SPEEK membrane was rigid as it exhibited lower *E_b_* compared to other fabricated membranes (Table 2). The pure SPEEK membrane showed lower water uptake which resulted to its lower flexibility [37]. Contrary, the pristine SPPO membrane showed higher water uptake than other fabricated membranes which resulted to its higher flexibility. From here, it was noticed that the introduction of SPPO into membrane matrix resulted to higher flexibility.

### 3.6. Thermal and Chemical Stability

**Figure 4:**
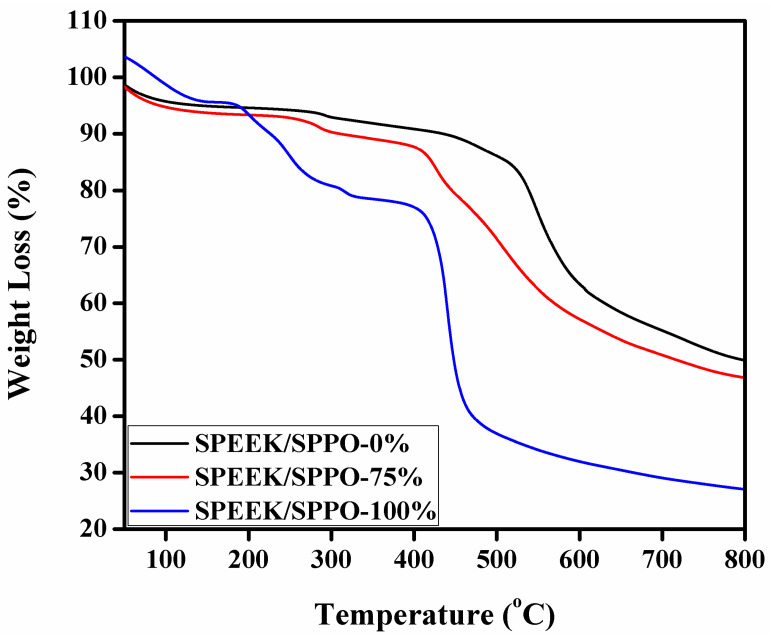
TGA thermograms of the fabricated blended membranes (where 0, 75 and 100% represent the amount of SPPO into the SPEEK/SPPO blended membranes).

It was measured by using thermogravimetric analyzer (TGA) and attained results are shown in Figure 4. According to the obtained results, all the prepared blended membranes underwent three step decomposition. The first decrease in mass at 90 to 130 °C occurred due to the water loss (free and bound water) as well as residual solvent [37,56]. The next loss in mass was observed at 180 to 340 °C due to pyrolysis of functional groups within the membranes matrix [42]. For pristine SPEEK membrane (SPEEK/SPPO-0%), the weight loss due to decomposition of functional group (-SO_3_H) began 285 °C as shown in Figure 4. The final mass loss was observed from 410 to 590 °C due to decomposition of the polymer backbone [57,58]. It proves that the blended membranes have enhanced thermal stability necessary for application in PEMFCs.

**Figure 5:**
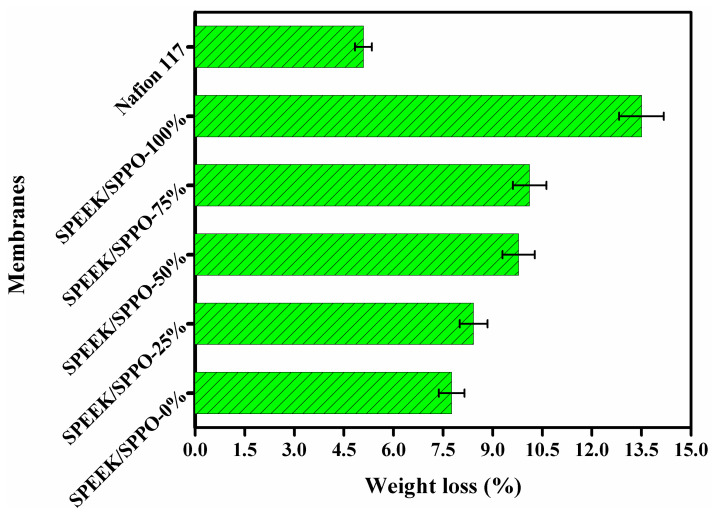
Weight loss of SPEEK, SPPO and SPEEK/SPPO blended membranes after immersion into Fenton’s reagent (3 wt% H_2_O_2_ and 3 ppm Fe^2+^) at 60 °C for two weeks (where 0, 25, 50, 75 and 100% represent the amount of SPPO into the SPEEK/SPPO blended membranes).

The chemical stability of the fabricated SPEEK, SPPO, and SPEEK/SPPO blended membranes was compared with commercial membrane Nafion 117 and determined in terms of weight loss after immersion into Fenton’s reagent. Figure 5 indicates the weight loss of the fabricated membranes and commercial membrane Nafion 117. Results showed that the membranes under investigation possessed higher chemical stability. Moreover, the color of membranes was unchanged after two weeks. The percentage weight loss was found to be varied from 7.76% to 13.50%. From Figure 5, it was noted that the fabricated SPPO (SPEEK/SPPO-100%) membrane showed maximum weight loss (only 13.50%). Contrary, the weight loss of commercial membrane was only 5.10%. The weight loss was gradually increased by increasing SPPO’s amount into the polymer matrix. We found that the maximum weight loss after two-week immersion was only about 13.50%. Results represented that these prepared membranes showed lower chemical stability than commercial membrane Nafion 117. However, the weight loss (13.50%) of the prepared membranes was after 2 weeks was very small which showed that the prepared membrane exhibited good chemical stability. It showed that the chemical stability of the fabricated membrane was sufficient for fuel cell application.

### 3.7. Proton Conductivity

**Figure 6:**
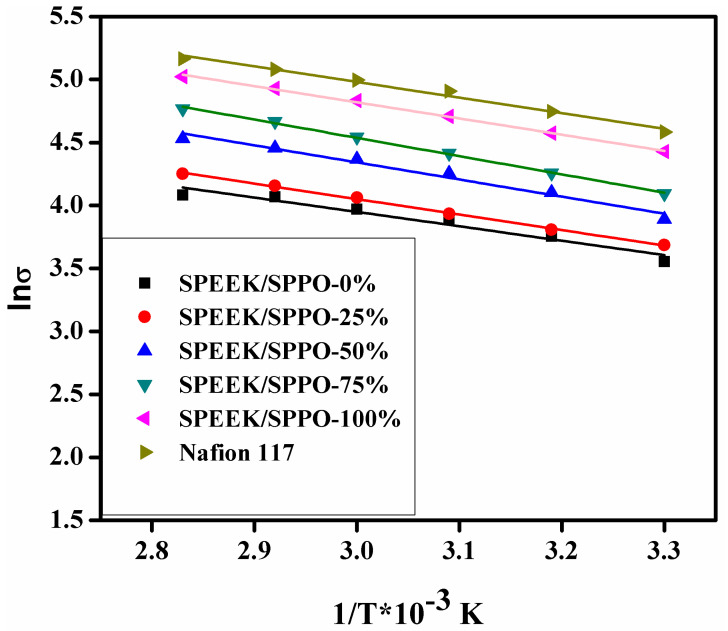
Arrhenius plot of proton conductivity of SPEEK, SPPO and blended SPEEK/SPPO membranes (where 0, 25, 50, 75 and 100% represent the amount of SPPO into the SPEEK/SPPO blended membranes).

The proton conductivities of the fabricated SPEEK, SPPO, blended SPEEK/SPPO membranes and commercial membrane Nafion 117 as function of temperature were recorded. Higher proton conductivity was observed in case of the fabricated blended membranes than the pristine SPEEK membrane. At 30 °C, the SPEEK, and SPPO membranes showed proton conductivities of 35 mS/cm, and 84 mS/cm, respectively. Contrary the fabricated blended membranes SPEEK/SPPO-25%, SPEEK/SPPO-50% and SPEEK/SPPO-75% represented proton conductivities of 40 mS/cm, 49 mS/cm and 60 mS/cm, respectively, at 30 °C. It was observed that by increasing the amount of SPPO into the polymer matrix, the proton conductivity was affected positively. It was due to increase in hydrophilicity of the prepared blended SPEEK/SPPO membranes. The value of proton conductivity for SPPO membrane was found to be similar to the already reported one in our previous work [59]. However, the obtained values of proton conductivities for the blended membranes were lower than commercial membrane Nafion 117 (98 mS/cm) at 30 °C, but these values of proton conductivities for the prepared blended membranes SPEEK/SPPO were notably higher and enough for development of fuel cell. Furthermore, the proton conductivity of the fabricated membranes appeared to be directly proportional to the temperature due to acceleration in mobility of proton. The further investigation of mechanism in the blended SPEEK/SPPO membranes was carried out by determining the activation energy values for proton conductivity as derived by the linear fitting of data as shown in Figure 6 by using Arrhenius type equation and the attained values of activation energy are given in Table 3.

$$\sigma =\sigma_{o}e^{-\frac{E_{a}}{RT}}$$

where *σ* is the pre-exponential factor, *E_a_* is the activation energy, *R* is the gas constant and *T* is the Kelvin temperature.

## 4. Conclusions

In this article, the fabrication of blended SPEEK/SPPO membranes for fuel cell application was performed via solution casting method. The synthesis of these blended membranes was verified by using FTIR spectroscopy. These fabricated blended membranes showed higher thermal, chemical and mechanical stability required for fuel cell application. Ion exchange capacity, water uptake and membrane swelling were increased by enhancing the concentration of SPPO into the membrane matrix. They showed homogeneous morphology. Results showed that proton conductivity increased from SPEEK/SPPO-0% (35 mS/cm) to SPEEK/SPPO-100% (84 mS/cm) when increasing the amount of SPPO within the polymer matrix. The pristine SPEEK membrane showed lower proton conductivity while pristine SPPO membrane higher proton conductivity. The proton conductivity of the prepared membranes was suitable for fuel cell application and showed an increasing trend with temperature due to increase in mobility of proton. Therefore, the low cost blended membranes SPEEK/SPPO compared to commercial membrane Nafion 117 were achieved for PEMFC application. From this, it was concluded that these fabricated membranes are the outstanding candidate for PEMFC.

## Figures and Tables

**Table 1:** Composition, ion exchange capacity, water uptake and swelling ratio of SPEEK/SPPO-0 to SPEEK/SPPO-100 membranes (where 0, 25, 50, 75 and 100% represent the amount of SPPO into the SPEEK/SPPO blended membranes).

Membranes	SPEEK (g)	SPPO (g)	*IEC* (mmol/g)	*W_R_* (%)	*MS* (%)
SPEEK/SPPO-0%	100	0	1.23	22.92	7.53
SPEEK/SPPO-25%	75	25	1.35	43.06	11.39
SPEEK/SPPO-50%	50	50	1.46	47.51	16.22
SPEEK/SPPO-75%	25	75	1.69	59.58	21.62
SPEEK/SPPO-100%	0	100	2.0	64.57	25.49

**Table 2:** Tensile strength and elongation at break of the fabricated membrane SPEEK/SPPO-0 to SPEEK/SPPO-100 (where 0, 25, 50, 75 and 100% represent the amount of SPPO into the SPEEK/SPPO blended membranes).

Membranes	Tensile Strength (MPa)	Elongation at Break (%)
SPEEK/SPPO-0%	14.36	3.89
SPEEK/SPPO-25%	7.58	6.79
SPEEK/SPPO-50%	14.27	8.00
SPEEK/SPPO-75%	11.48	11.35
SPEEK/SPPO-100%	20.42	41.91

**Table 3:** Activation Energy of the fabricated membranes SPEEK/SPPO-0 to SPEEK/SPPO-100 (where 0, 25, 50, 75 and 100% represent the amount of SPPO into the SPEEK/SPPO blended membranes).

Sr. No.	*E_a_* (eV)
SPEEK/SPPO-0%	9.49
SPEEK/SPPO-25%	10.31
SPEEK/SPPO-50%	11.29
SPEEK/SPPO-75%	12.05
SPEEK/SPPO-100%	10.68
Nafion 117	10.60

## Data Availability

Not applicable.

## Nomenclature

Codes	Full names
IEM	Ion exchange membranes
PEM	Proton exchange membrane
SPEEK	Sulfonated poly(ether ether ketone)
SPPO	Sulfonated poly(phenylene) oxide
